# Supplementary material for: BMP-9 mediates fibroproliferation in fibrodysplasia ossificans progressiva through TGF-β signaling
Source: EMBO Mol Med. 2024 Dec 3;17(1):112–28. doi: 10.1038/s44321-024-00174-3 (PMC11729865; doi:10.1038/s44321-024-00174-3)
Supplement: Supplementary file 1 — Appendix [file 44321_2024_174_MOESM1_ESM.pdf]

Appendix

BMP-9 mediates fibroproliferation in fibrodysplasia ossificans progressiva through TGF-β signaling

Chengzhu Zhao[1,2,*], Yoshiko Inada[2], Souta Motoike[2], Daisuke Kamiya[2,3], Kyosuke Hino[2,4], and Makoto Ikeya[2,3,*]

*To whom correspondence may be addressed: Chengzhu Zhao, Makoto Ikeya,

Email: chengzhu.zhao@cqmu.edu.cn, mikeya@cira.kyoto-u.ac.jp

Table of Contents

Appendix Figure S1……………………………………………………………………………2

Appendix Figure S2……………………………………………………………………………3

Appendix Figure S3……………………………………………………………………………4

Appendix Figure S4……………………………………………………………………………5

Appendix Figure S5……………………………………………………………………………6

Appendix Figure S6……………………………………………………………………………8

Appendix Figure S7……………………………………………………………………………10

Appendix Figure S8……………………………………………………………………………11

Appendix Figure S9……………………………………………………………………………12

Appendix Table S1 ……………………………………………………………………………13

Appendix Table S2 ……………………………………………………………………………15

Appendix Table S3 ……………………………………………………………………………16

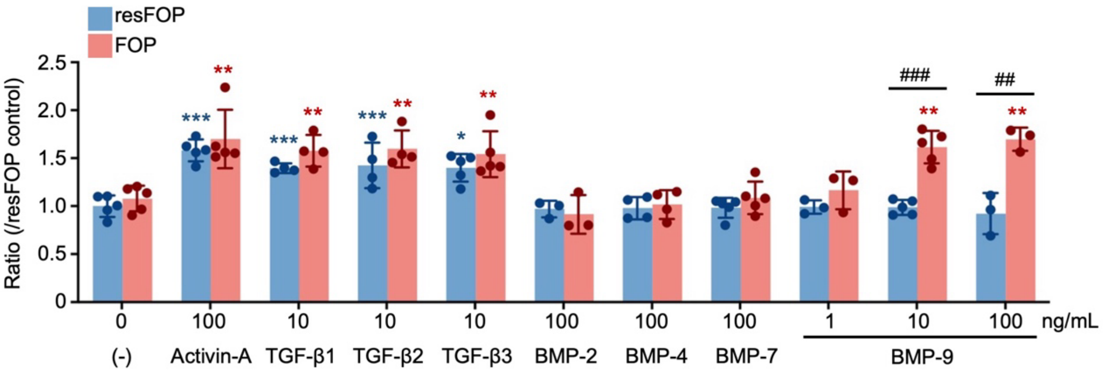

Appendix Figure S1. Detailed data of FOP-ACVR1-specific ligand screening. resFOP- and FOP-iMSCs were treated with a series of TGF-β superfamily members. After 72 h of incubation, the cell proliferation ratio was measured using the CCK-8 assay. The results represent the mean ± SD (n = 3-6). *$P < 0.05$; **$P < 0.01$; ***$P < 0.001$ compared to the no ligand treatment control; ##$P < 0.01$; ###$P < 0.001$ compared to resFOP-iMSCs treated with the same ligands, analyzed using two-way ANOVA with Tukey's multiple comparisons.

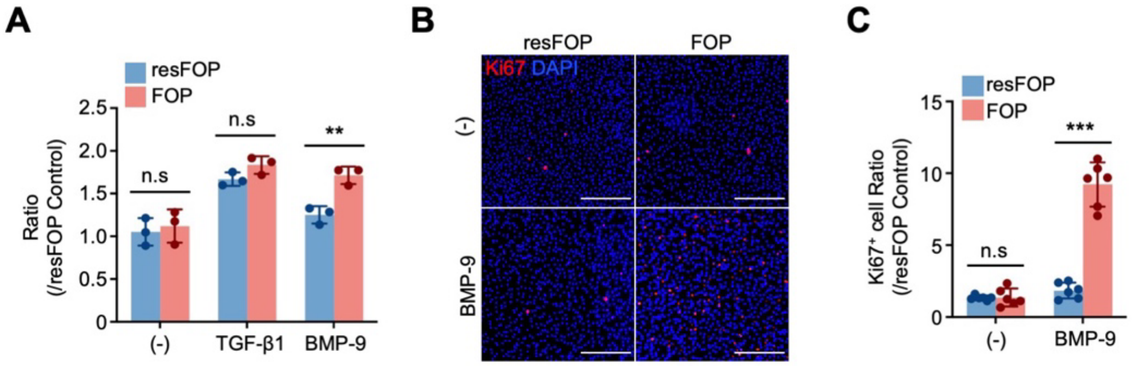

Appendix Figure S2. BMP-9 specifically activates the proliferation of FOP-iMSCs derived from another patient, but not resFOP-iMSCs. (A) Cell proliferation rates of FOP- and resFOP-iMSCs were tested by the CCK-8 assay. BMP, 100 ng/mL BMP-7; TGF, 10 ng/mL TGF-β1. Results represent the mean ± SD (n = 3). n.s., no significant difference; ***P* < 0.01 by multiple *t*-tests in comparison with resFOP-iMSCs under the same condition. (*B*) Representative images of Ki67 immunofluorescent staining. Scale bar, 100 μm. (*C*) Quantitative analysis of the ratio of Ki67^{+} cells. Data represent the mean ± SD (n = 6). 6 randomly selected panels were analyzed, with at least 4,710 cells counted per panel. n.s., no significant difference; ****P* < 0.001 by multiple *t*-tests in comparison with resFOP-iMSCs under the same condition.

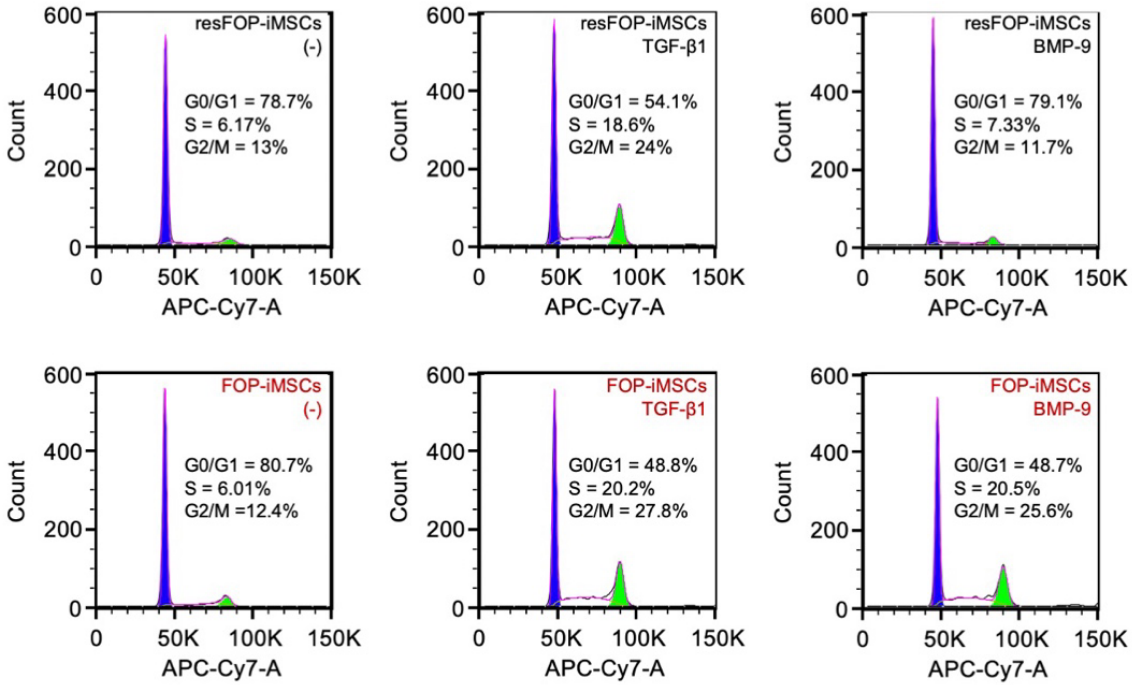

Appendix Figure S3. Representative flow cytometry plots. FOP- and resFOP-iMSCs were treated with TGF-β1 (10 ng/mL) or BMP-9 (100 ng/mL) for 72 h and then stained with a Cell Cycle Assay Kit.

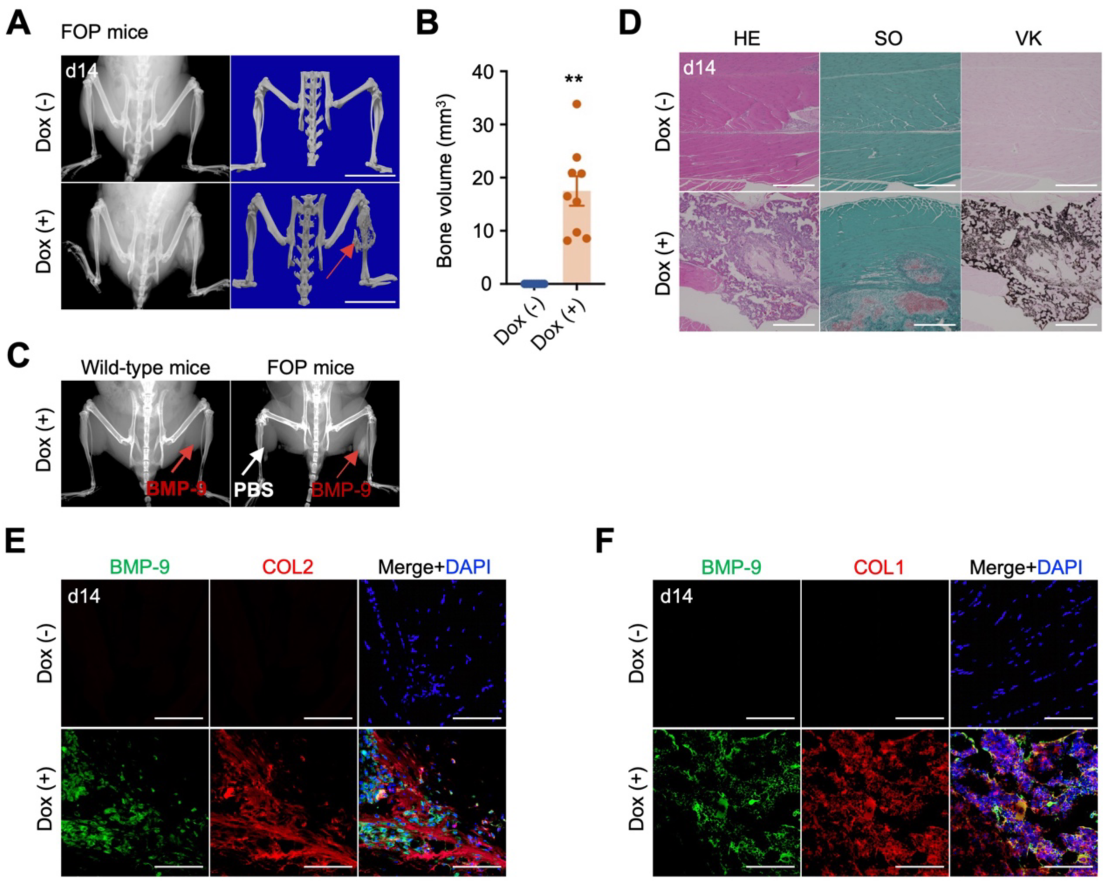

Appendix Figure S4. Histological analysis of the BMP-9-injected region (day 14). (*A*) Representative X-ray and μCT observations. Scale bar, 10 mm. (*B*) Heterotopic bone volume (mm^3) in each group. The results represent the mean ± SD (n = 9). ***P* < 0.01 by Student's *t*-test in comparison with the Dox (-) group. (*C*) Representative X-ray observations of wild-type mice receiving Dox and BMP9 (n = 6), and Dox (+) FOP mice with PBS injection. (*D*) H&E, safranin O, and von Kossa staining (calcium). Scale bars, 500 μm. (*E* and *F*), Anti-BMP-9, anti-COL2 (*E*), and anti-COL1 (*F*) staining. Scale bars, 100 μm.

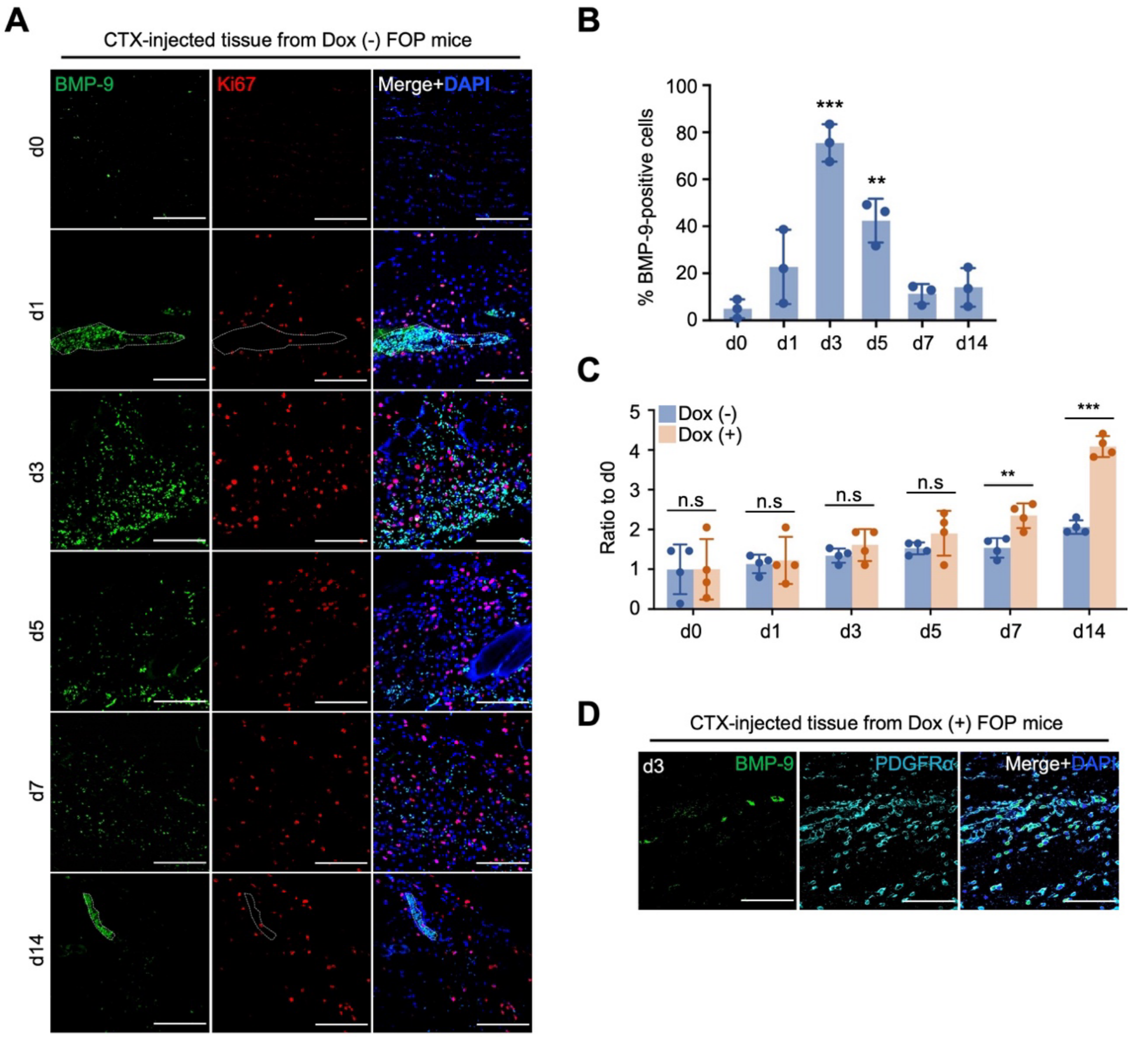

Appendix Figure S5. Tissue distribution and serum levels of BMP-9 in CTX-treated FOP mice. (*A*) Immunohistochemical staining for BMP-9 (green) and Ki67 (red) at various time points (d1, 3, 5, 7, and 14) in CTX-injected Dox (-) FOP mice. Scale bar, 100 µm. (*B*) Percentage of BMP-9-positive cells relative to the total number of DAPI-stained nuclei. The BMP-9-positive cell clusters outlined by white dashed lines were excluded from the quantification. The results represent the mean ± SD (n = 3). 3 randomly selected panels were analyzed, with an average of 260 cells counted per panel. ****P* < 0.001 by one-way ANOVA with Dunnett's multiple comparisons test compared to the CTX-untreated group (d0). (*C*) Circulating BMP-9 levels in CTX-injected Dox (-) and Dox (+) FOP mice. The results represent the mean ± SD (n = 4). n.s., no significant difference; ***P* < 0.01, ****P* < 0.001 by multiple *t*-tests in comparison with the Dox (-) FOP mice group at the same time points. (*D*) Co-

immunofluorescence staining of BMP-9 (green) with PDGFRα^{+} cells (light blue) at day 3 post-CTX injection in FOP tissue. Scale bar, 50 μm.

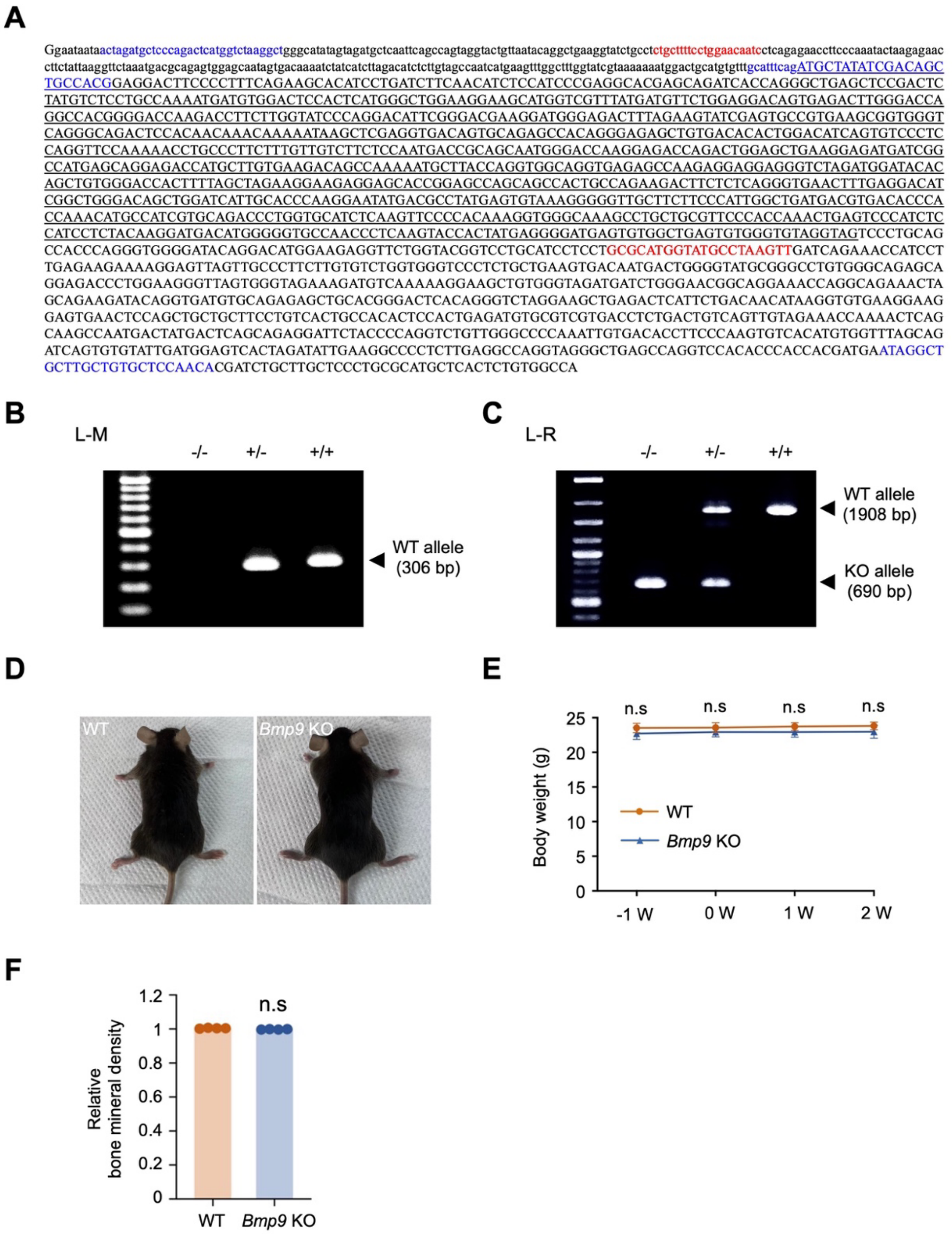

Appendix Figure S6. Generation and subsequent observations for *Bmp9*-KO-*hFOP-ACVR1* mice. (*A*) Targeted gene sequence: sequences targeted by single-guide RNAs are marked in red; *Bmp9* PCR primers are marked in blue. The sequence of exon 2 is underlined, while the 3′ UTR is indicated by a dashed underline. (*B* and *C*) Genotyping PCR of *Bmp9*-KO-*hFOP-ACVR1* transgenic

mice. (*B*) Gel electrophoresis shows the deletion (mutant) and WT bands (306 bp) when using PCR primers allocated to the left genomic region (L; left) and deleted region (M; middle) of the gRNAs. (*C*) Using primers allocated to the left genomic region (L; left) and the right region (R; right) of the gRNAs, the mutant allele (690 bp) and the WT allele (1908 bp) are shown. (*D*) Representative images of 16-week-old adult mice from the experimental groups. (*E*) Body weight of WT-*hFOP-ACVR1* and *Bmp9*-KO-*hFOP-ACVR1* mice. The results represent the mean ± SD (n = 4). n. s., no significant difference by multiple *t*-tests in comparison with the WT-*hFOP-ACVR1* mouse group at the same time points. (*F*) Relative heterotopic bone mineral density of each group. The results represent the mean ± SD (n = 4). n.s., no significant difference by Student's *t*-test in comparison with the WT-*hFOP-ACVR1* group.

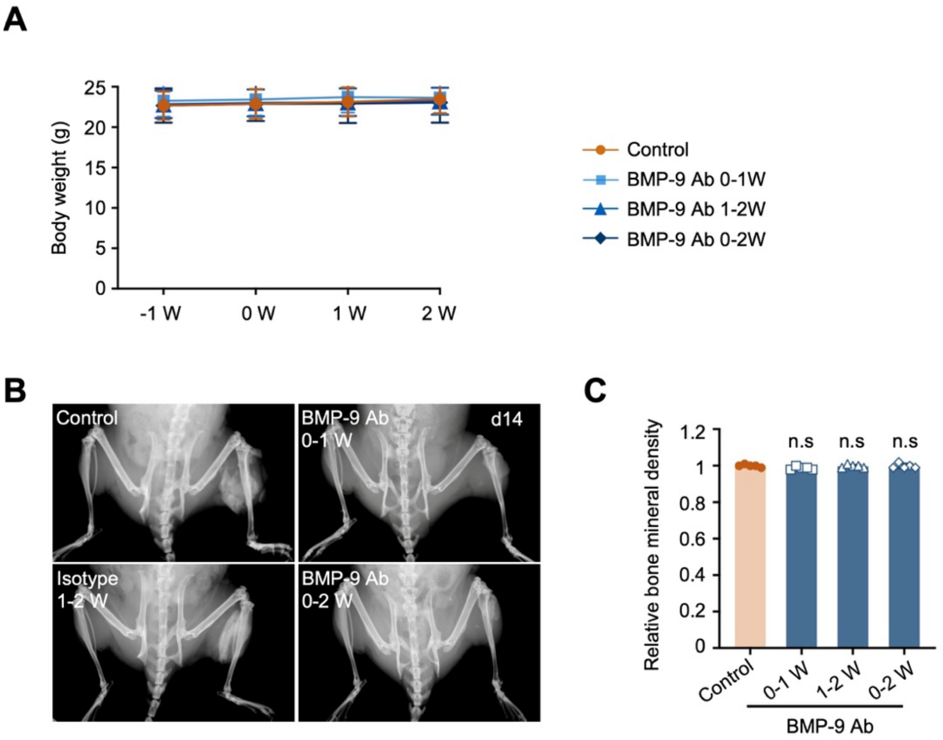

Appendix Figure S7. BMP-9 neutralizing antibody suppressed CTX-induced HO. Female C57BL/6 mice (8-12 w) were treated with 10 mg/kg BMP-9 neutralizing antibody (BMP-9 Ab) or IgG2B isotype control antibody (control) subcutaneously twice a week from the day of CTX injection for 1 week (0-1 W), 2 weeks (0-2 W), or 1 week after CTX injection for 1 week (1-2 W). (*A*) Body weight of isotype control and BMP-9 Ab administrated FOP-ACVR1 conditional transgenic mice. The results represent the mean ± SD (n = 5). n. s., no significant difference in comparison with the control antibody-treated group at the same time points, analyzed using two-way ANOVA with Tukey's multiple comparisons. (*B*) Representative radiographic images of the experimental groups. (*C*) Relative heterotopic bone mineral density of each group. The results represent the mean ± SD (n = 5). n.s., no significant difference by one-way ANOVA with Dunnett's multiple comparisons test compared to the control antibody-treated group.

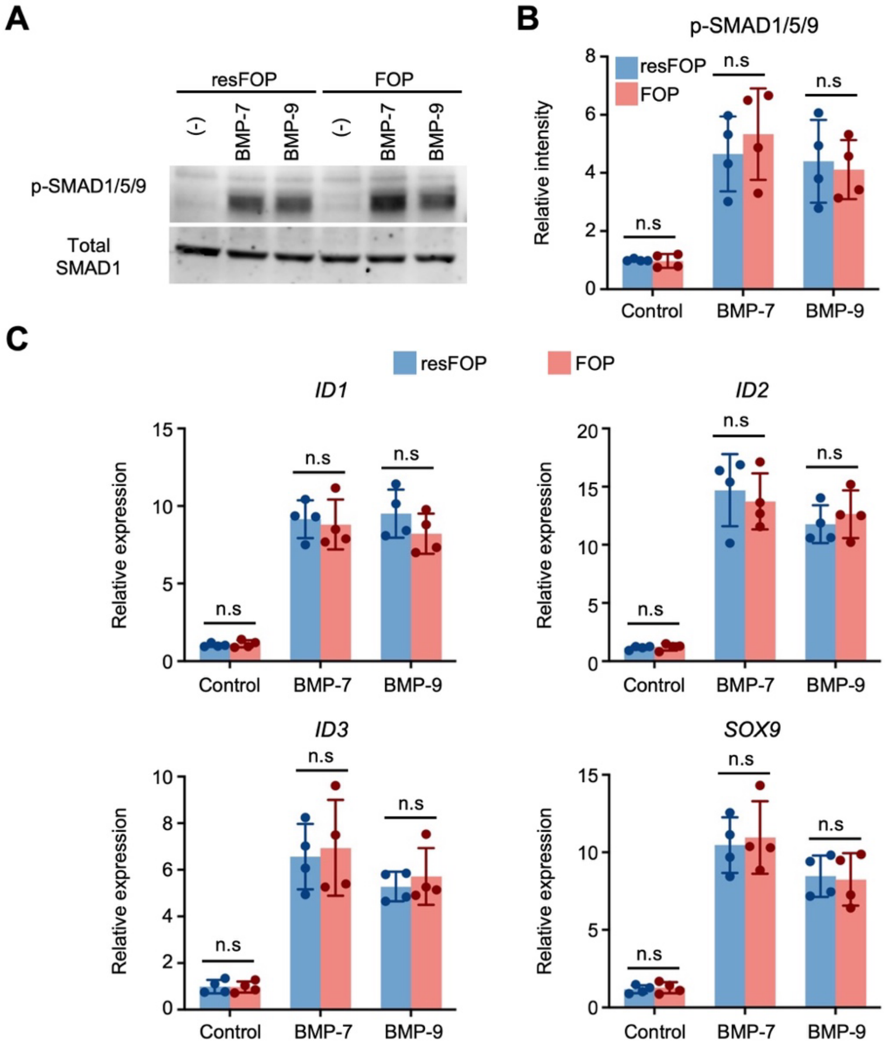

Appendix Figure S8. BMP-9 transduced BMP-SMAD1/5/9 signaling similarly in FOP- and resFOP-iMSCs. (*A*) Representative image of western blot analysis. BMP-9 induced phosphorylation of SMAD1/5/9 (p-SMAD1/5/9) similarly in FOP- and resFOP-iMSCs. (*B*) Quantification of relative phosphorylation levels of SMAD1/5/9. (*C*) Expression levels of BMP target genes in FOP-iMSCs stimulated with BMP-9 in quantitative PCR analysis. The results represent the mean ± SD (n = 4). n.s., no significant difference by multiple *t*-tests in comparison with resFOP-iMSCs under the same condition treated with the same ligand (B and C).

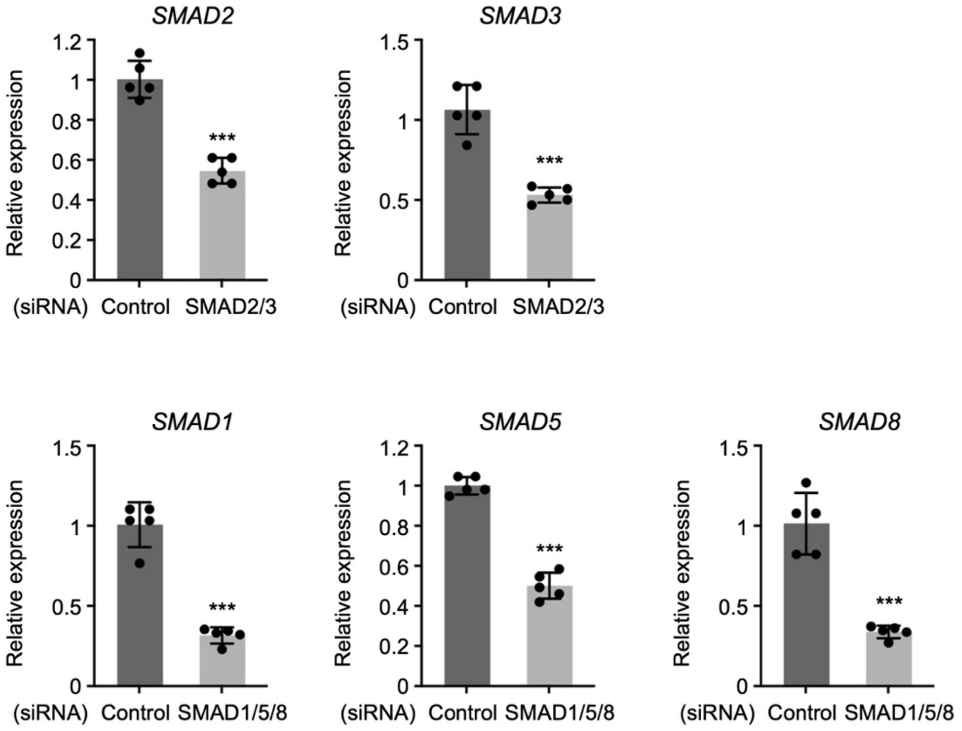

Appendix Figure S9. Knock-down efficiencies of siRNAs. FOP-iMSCs transiently transfected with siRNAs specific for SMADs were incubated for 16 h, and RNA was analysed. The results represent the mean ± SD (n = 5). ***P < 0.001 by Student's *t*-test compared to control siRNA-treated FOP-iMSCs.

Appendix Table S1. Antibodies for western blotting and immunostaining.

	Name	Company	Cat. No	Concentration
1st antibody	Ki-67 (D3B5) Rabbit mAb	Cell Signaling	#12202	1:500
	BMP-9 (H-3) Mouse mAb	Santa Cruz Biotechnology	sc-514211	1:100
	Collagen II Ab-2 (Clone 2B1.5) Mouse mAb	Thermo Fisher Scientific	#MS-235-B0	1:100
	Collagen I alpha 1 Antibody	Novus Biologicals	NB600-408	1:100
	Anti-F4/80 antibody [CI:A3-1]	abcam	ab6640	1:100
	Anti-PDGFR alpha antibody [RM0004-3G28]	abcam	ab51875	1:100
	Anti-SP7/Osterix antibody	abcam	ab22552	1:100
	Phospho-Smad2 (Ser465/467)/ Smad3 (Ser423/425) (D27F4) Rabbit mAb	Cell Signaling Technology	#8828	1:1000
	Smad2/3 (D7G7) XP® Rabbit mAb	Cell Signaling Technology	#8685	1:1000
	Phospho-Smad1 (Ser463/465)/ Smad5 (Ser463/465) / Smad9 (Ser465/467) (D5B10) Rabbit mAb	Cell Signaling Technology	#13820	1:1000
	Smad1/5/8 Antibody (N-18)-R	Santa Cruz Biotechnology	sc-6031-R	1:1000
2nd antibody	Anti-rabbit IgG, HRP-linked Antibody	Cell Signaling Technology	#7074	1:10000
	Goat anti-Mouse IgG (H+L) Secondary Antibody, Alexa Fluor® 488 conjugate	Invitrogen	A-28175	1:500
	Goat anti-Rabbit IgG (H+L) Cross-Adsorbed Secondary Antibody, Alexa Fluor® 555 conjugate	Invitrogen	A-21428	1:500
	Goat anti-Mouse IgG (H+L) Cross-Adsorbed Secondary Antibody, Alexa Fluor® 555	Invitrogen	A-21422	1:500

	Goat anti-Rat IgG (H+L) Cross-Adsorbed Secondary Antibody, Alexa Fluor® 555	Invitrogen	A-21434	1:500
	Goat anti-Rat IgG (H+L) Cross-Adsorbed Secondary Antibody, Alexa Fluor® 647	Invitrogen	A-21247	1:500

Appendix Table S2. Detailed information on the siRNAs used in the study.

Gene Symbol	siRNA assay ID	Catalog #
ACVRL1	s987	4392420
ACVR1	s976	4390824
BMPR1A	s282	4392420
ACVR1B	s977	4390824
TGFBR1	s229438	4392420
BMPR1B	s2041	4392420
ACVR1C	s43497	4392420
ACVR2A	s981	4390824
ACVR2B	s985	4390824
BMPR2	s2046	4390824
AMHR2	s1325	4392420
TGFBR2	s14078	4390824
SMAD2	s8397	4392420
SMAD3	s8400	4392420
SMAD1	s8934	4392420
SMAD5	s8406	4392420
SMAD9	s8415	4392420

Appendix Table S3. Primer sequences for qPCR analysis.

Gene	Forward	Reverse
ACTB	CACCATTGGCAATGAGCGGTTC	AGGTCTTTGCGGATGTCCACGT
CMYC	AGCGACTCTGAGGAGGAACA	TCGCCTCTTGACATTCTCCT
CTGF	CCTGTGCAGCATGGACGTT	GGACCAGGCAGTTGGCTCTAA
PMEPA1	CATGATCCCCGAGCTGCT	TGATCTGAACAAACTCCAGCTCC
CCL2	ATGAAAGTCTCTGCCGCCCTTCTGT	AGTCTTCGGAGTTTGGGTTTGCTTG
ID1	CCAACGCGCCTCGCCGGATC	CTCCTCGCCAGTGCCTCAG
ID2	AGTCCCGTGAGGTCCGTTAG	AGTCGTTCATGTTGTATAGCAGG
ID3	CTGGACGACATGAACCACTG	GTAGTCGATGACGCGCTGTA
CDK1	AAACTACAGGTCAAGTGGTAGCC	TCCTGCATAAGCACATCCTGA
CDK2	ATCCGCCTGGACACTGAGACT	TGGAGGACCCGATGAGAATG
CCNA	GCATGTCACCGTTCCTCCTTG	GGGCATCTTCACGCTCTATTTT
CCNB1	TTGGTTTCTGCTGGGTGTAGG	CCATGTTGATCTTCGCCTTATTT
BRCA2	TGGGCTCTCCTGATGCCTGTA	GTATACCAGCGAGCAGGCCG
CDKN2C	TGATCGTCAGGACCCTAAAG	TGATCGTCAGGACCCTAAAG
TP53	CGTGCAAGTCACAGACTTGGCTGTC	ACGGTGACACGCTTCCCTGGAT
CSK1B	GGACAAATACGACGACGAGGA	CTGACTCTGCTGAACGCCAAG
SMAD2	GAATTTGCTGCTCTTCTGGCTCAG	GCCATAGGGACCACAACACAATG
SMAD3	GAGGGCAGGCTTGGGGAAAATG	GGGAGGGTGCCGGTGGTGTAATAC
SMAD1	CCCAGGGCAACCGAGTAAC	TTGCAGCCTGCCATCCA
SMAD5	CAGGAAGGTCTCCGAAGATTTGT	CGCAGCCAGGCAAGTTG
SMAD9	CGCAGCCAGGCAAGTTG	AGTTGCGAAGTGTGTTGACTTTCT
