# Supplementary figures and images for: BMP-9 mediates fibroproliferation in fibrodysplasia ossificans progressiva through TGF-β signaling
Source: EMBO Mol Med. 2024 Dec 3;17(1):112–28. doi: 10.1038/s44321-024-00174-3 (PMC11729865; doi:10.1038/s44321-024-00174-3)

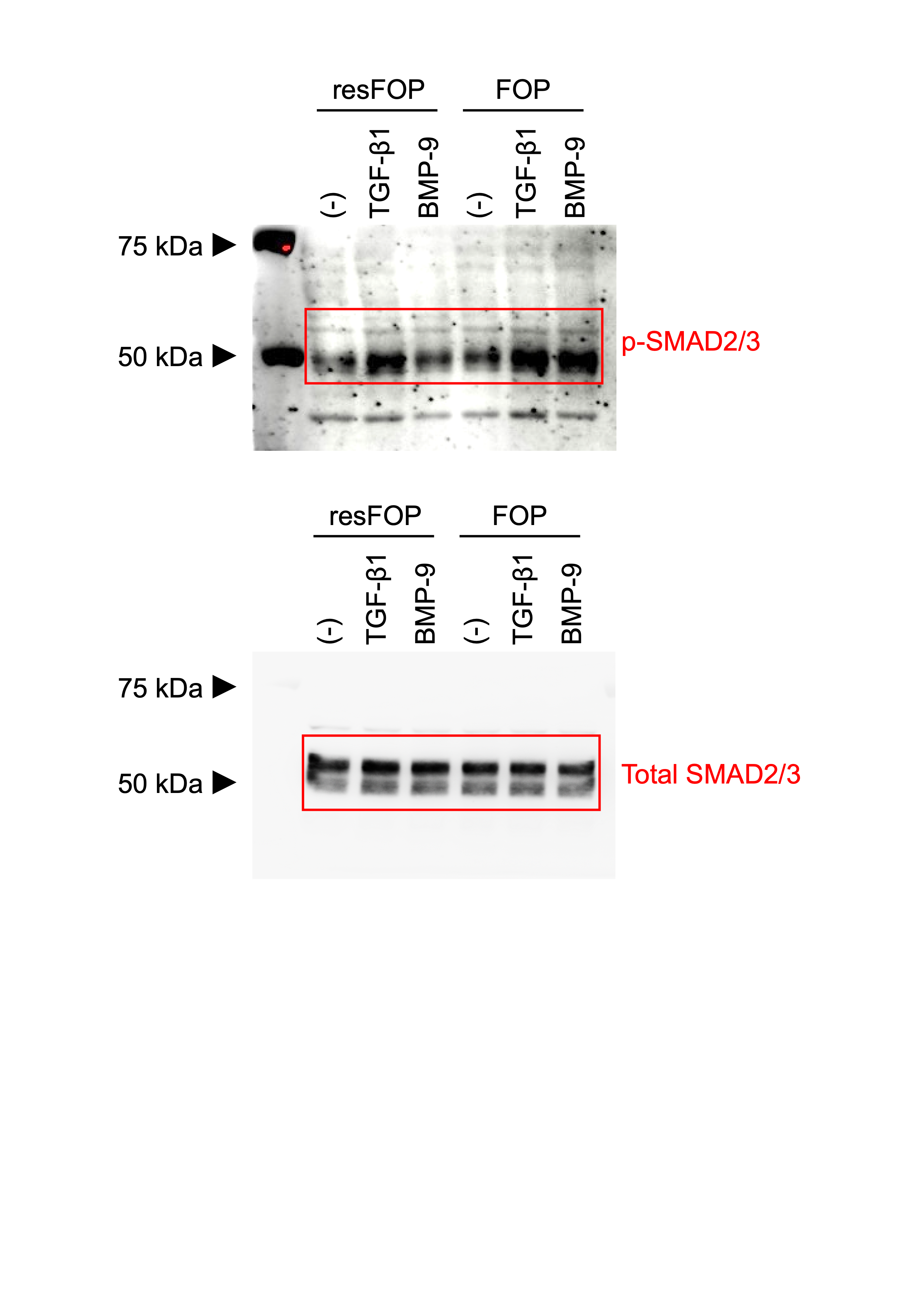

Supplement: Supplementary file 7 — Source data Fig. 5 [file 44321_2024_174_MOESM7_ESM.zip › Figure 5/5H Image and Numerical data/WB blots of Fig.5H.tiff]
